# The identification and structural analysis of potential 14-3-3 interaction sites on the bone regulator protein Schnurri-3

**DOI:** 10.1107/S2053230X21006658

**Published:** 2021-07-28

**Authors:** Lorenzo Soini, Seppe Leysen, Tom Crabbe, Jeremy Davis, Christian Ottmann

**Affiliations:** a Laboratory of Chemical Biology, Department of Biomedical Engineering and Institute for Complex Molecular Systems, Eindhoven University of Technology, Eindhoven, The Netherlands; b Department of Chemistry, UCB Celltech, Slough, United Kingdom; c Department of Structural Biology and Biophysics, UCB Celltech, Slough, United Kingdom; d New Targets, UCB Celltech, Slough, United Kingdom

**Keywords:** bone regulator protein, Schnurri-3, X-ray protein crystallography, phosphorylation, fluorescence polarization, disulfide bonds, 14-3-3 modes

## Abstract

14-3-3 proteins regulate many intracellular processes and their ability to bind in subtly different fashions to their numerous partner proteins provides attractive drug-targeting points for a range of diseases. Schnurri-3 is a suppressor of mouse bone formation and a candidate target for novel osteoporosis therapeutics, and thus it is of interest to determine whether it interacts with 14-3-3. In this work, potential 14-3-3 interaction sites on mammalian Schnurri-3 were identified by an *in silico* analysis of its protein sequence. Using fluorescence polarization, isothermal titration calorimetry and X-ray crystallography, it is shown that synthetic peptides containing either phosphorylated Thr869 or Ser542 can indeed interact with 14-3-3, with the latter capable of forming an interprotein disulfide bond with 14-3-3σ: a hitherto unreported phenomenon.

## Abstract 2

The structures of two peptide motifs of Schnurri-3 in complex with 14-3-3 are reported.

## 1. Introduction   

14-3-3 proteins are a family of regulatory adaptor proteins with an interactome currently estimated to include almost 500 binding partners (Aitken, 2006 ▸). They facilitate cellular processes such as signal transduction, cellular trafficking, apoptosis and cell-cycle regulation (Hermeking & Benzinger, 2006 ▸; Mackintosh, 2004 ▸; Johnson *et al.*, 2011 ▸). 14-3-3 proteins are expressed in all eukaryotic cells and human compartments, reaching a remarkable percentage of 1% of soluble brain proteins (Cornell & Toyo-Oka, 2017 ▸). In humans, seven isoforms (β, γ, σ, ζ, η, ɛ and τ) of 14-3-3 exist. They share a high degree of homology and are known to form homodimers and heterodimers. Structurally, each 14-3-3 monomer is formed by nine α-helices connected by short loops, which confers a high degree of rigidity when docking onto binding partners (Sluchanko & Gusev, 2010 ▸; Obsil & Obsilova, 2011 ▸).

The binding of 14-3-3 proteins to their protein partners occurs via the recognition of specific consensus motifs that includes a phosphorylated serine or threonine. To date, three of these motifs have been defined: mode I, RS*X*pS/pT*X*P, mode II, R*X*Y/F*X*pS/pT*X*P, and mode III, pS/pT*X*_1–2_-COOH (Yaffe *et al.*, 1997 ▸). *X* represents any residue and mode III motifs are situated in the C-terminus of the binding partner. However, many exceptions to these motifs have also been identified (Johnson *et al.*, 2010 ▸). Tens of crystal structures of 14-3-3 proteins bound to these motifs in the form of synthetic phosphorylated peptides have been deposited in the PDB (Sluchanko, 2018 ▸), and together they provide an important resource that can be exploited for the understanding and modulation of 14-3-3 protein–protein interactions (PPIs).

Regulation of osteoblast activity is essential for the preservation of bone homeostasis (Harada & Rodan, 2003 ▸). Schnurri-3 (SHN3), also known as transcription factor HIVEP3, has been identified as a key inhibitory regulator of osteoblast activity in the context of mouse postnatal skeletal remodelling (Jones *et al.*, 2007 ▸). It has been proposed that this is achieved via interaction with the MAPK ERK1/2 and suppression of the kinase activity critical to pro-osteogenic signalling pathways (Shim *et al.*, 2013 ▸; Kim *et al.*, 2019 ▸), although it remains unclear how MAPK or pathway specificity is achieved. Mice lacking critical residues in the MAPK-binding motif of SHN3 or mice treated to reduce the expression of endogenous SHN3 nevertheless displayed few phenotypic changes beyond increased adult bone mass, identifying SHN3 as an attractive drug target for osteoporosis and fracture-repair treatments (Yang *et al.*, 2019 ▸).

The interaction of SHN3 with kinases opens up the possibility that its function is more specifically regulated by phosphorylation and binding to 14-3-3 proteins. To initiate investigations, we performed an *in silico* analysis and found 18 potential 14-3-3 interaction sites in human SHN3, only two of which were conserved in the mouse sequence. Phosphorylated synthetic peptides of these two consensus motifs were found to interact with 14-3-3 *in vitro* and their binding modes were further characterized by X-ray crystallography. Of particular interest, we observed that the motif built around Ser542 in human SHN3 can become covalently associated with 14-3-3σ via an interprotein disulfide bond.

## 2. Materials and methods   

### 2.1. Protein expression and purification   

14-3-3 isoforms γ, τ, σ, ɛ, η, ζ, β (full length) and 14-3-3σΔC (residues 231–248 deleted) were expressed with a His_6_ tag in *Escherichia coli* NiCo21(DE3) competent cells from a pPRoeX-Htb vector in 2×TY medium. Purification was carried out by affinity chromatography on nickel columns (HisTrap HP, 5 ml). The tags were cleaved with TEV protease. The proteins were then loaded again onto nickel columns to remove any noncleaved protein. A final purification step was performed by loading the proteins onto a size-exclusion chromatography column (HiLoad 26/600 Superdex 75 pg) equilibrated in 20 m*M* Tris–HCl pH 7.5, 150 m*M* NaCl, 2 m*M* DTT. All purification steps were performed on an ÄKTApure protein-purification system (Cytiva). The full-length 14-3-3 isoforms were used in biophysical assays and the 14-3-3σΔC construct was used for crystallographic purposes.

The peptides SHN3pS542 (LLRSHpS542MPSAAC) and SHN3pT869 (PDRPDpT869EPEPPP) were ordered with a purity of >95% from GenScript in an N-terminally acetylated version and an N-terminally FITC-Ahx-labelled version.

### 2.2. Fluorescence polarization and isothermal titration calorimetry   

Fluorescence polarization (FP) binding assays were carried out in Corning 384-well 3575 plates, serially diluting (twofold) the 14-3-3 isoforms in the presence of 10 n*M* FITC-labelled peptides. Proteins and peptides were diluted from their stock concentration in assay buffer [50 m*M* Tris pH 7.5, 150 m*M* NaCl, 5 m*M* MgCl_2_, 0.05%(*v*/*v*) Tween-20]. The data were collected on a PHERAstar FSX plate reader (BMG Labtech) with an excitation wavelength of 485 nm and an emisson wavelength of 520 nm. For *K*_d_ calculation, the background polarization was removed from all values and the data were fitted with a one-site specific binding model in *GraphPad Prism* version 8.1.1 for Windows (GraphPad Software, La Jolla, California, USA; https://www.graphpad.com). Each data point is the average of a triplicate measurement; the standard deviation is reported as error bars. ITC measurements were performed on a PEAQ-ITC (Malvern), dissolving the peptides in assay buffer [50 m*M* Tris pH 7.5, 150 m*M* NaCl, 5 m*M* MgCl_2_, 2 m*M* 2-mercaptoethanol, 0.05%(*v*/*v*) Tween-20] and dialysing the protein in the same buffer to minimize buffer mismatch. The optimal peptide and protein concentrations were chosen by simulating the experiment using the MicroCal PEAQ-ITC Analysis Software (Malvern) according to the predicted *K*_d_. The peptides were titrated into a cell containing the protein using a series of 18 injections of 2 µl each at 25°C (reference power 5 µcal s^−1^, stirring speed 750 rev min^−1^, initial delay 60 s, spacing 150 s). The data were analysed using the MicroCal PEAQ-ITC Analysis Software (Malvern).

### 2.3. X-ray protein crystallography   

For protein crystallization, a C-terminally truncated version of 14-3-3σ was used (14-3-3σΔC; deletion of the 17 C-terminal amino acids; Schumacher *et al.*, 2010 ▸). The binary complexes were prepared by mixing 14-3-3σΔC at 10–15 mg ml^−1^ with SHN3pS542 and SHNpT869 in 1:1 and 1:1.2 molar ratios, followed by overnight incubation at 4°C in dilution buffer (20 m*M* HEPES pH 7.5, 2 m*M* MgCl_2_). The crystallization drops were dispensed in a 1:1 protein solution:crystallization condition ratio (0.5 µl + 0.5 µl). A focused set of crystallization conditions was used consisting of 95 m*M* HEPES pH 7.1–7.7, 24–29% PEG 400, 190 m*M* CaCl_2_, 5% glycerol. The plate was incubated at 4°C; crystals grew in 5–10 days and were observed in most of the crystallization conditions. The data were collected on the I04 beamline at Diamond Light Source. The diffraction data were processed with *XDS* (Kabsch, 2010 ▸) and *AIMLESS* (Evans, 2011 ▸; Winn *et al.*, 2011 ▸). The structure was solved by molecular replacement using *Phaser* (McCoy *et al.*, 2007 ▸) with a 14-3-3σ structure as a search model (PDB entry 3mhr; Schumacher *et al.*, 2010 ▸). The initial structure was then refined using *Coot* (Emsley *et al.*, 2010 ▸) and *Phenix* (Liebschner *et al.*, 2019 ▸).The structures have been deposited in the PDB with accession codes 7b13 for 14-3-3σ–SHN3pS542 and 7b15 for 14-3-3σ–SHN3pT869. All figures were generated using *PyMOL* (version 1.2r3pre; Schrödinger).

## 3. Results and discussion   

### 3.1. *In silico* analysis of the SHN3 protein sequence   

To identify potential 14-3-3 binding sites on SHN3 (UniProt ID Q5T1R4), we performed a sequence analysis using the *14-3-3-Pred* web server (Madeira *et al.*, 2015 ▸). Of all of the serine and threonine residues identified within the SHN3 sequence, 18 scored the highest values for selection as a potential 14-3-3 binding site (Table 1 ▸). Among these 18, we focused our attention on those that more closely resembled the mode I and mode II 14-3-3 binding motifs, with an arginine at position −3/−4 and a proline at position +2 from the serine/threonine that is phosphorylated. Particular focus has been given to Pro +2, which has been reported to be of crucial importance for 14-3-3 recognition (Yaffe *et al.*, 1997 ▸; Rittinger *et al.*, 1999 ▸; Sluchanko & Gusev, 2010 ▸). Following this strategy, a more detailed analysis was performed on the sites Ser542, Thr869, Ser1894, Thr1401 and Thr2339. Since sequence conservation in different species often correlates with biological function, an alignment of the human and mouse (*Mus musculus*) SHN3 sequences was performed. This strategy also prioritizes motifs that can be investigated for function in genetically engineered model organisms. The alignment revealed that only Ser542 and Thr869 were conserved in mouse SHN3, and therefore we concentrated our *in vitro* experiments on these sites.

### 3.2. Biophysical characterization of the binding of SHN3pS542 and SHN3pT869 peptides to 14-3-3 proteins   

To investigate potential 14-3-3 interaction sites on a given protein, short synthetic phosphorylated peptides derived from their native sequences can be studied in biophysical assays. This circumvents the challenges associated with producing full-length proteins, phosphorylated at specific sites, in sufficient amounts and with sufficient purity. Several examples employing this approach have been reported in the literature (Ballone *et al.*, 2018 ▸; Centorrino *et al.*, 2018 ▸; Rose *et al.*, 2012 ▸). Two peptides derived from the SHN3 protein sequence were designed: SHN3pS542 (LLRSHpS542MPSAAC) and SHN3pT869 (PDRPDpT869EPEPPP).

**Figure 1:**
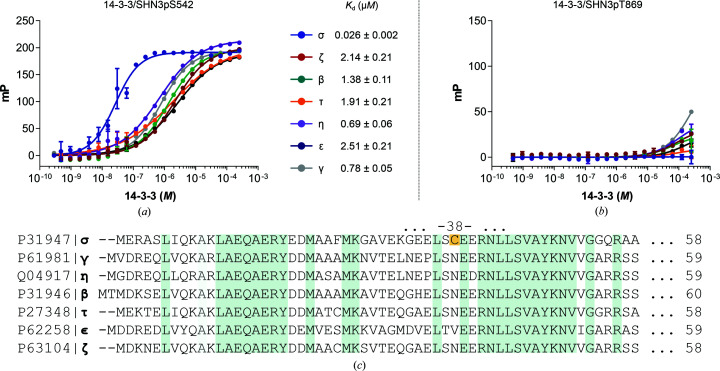
Fluorescence polarization assays of the SHN3 peptides with all human 14-3-3 isoforms. (*a*, *b*) Titration curves of 14-3-3 proteins titrated against a constant concentration of SHN3 peptide. *K*_d_ values are reported on the right for the SHN3pS542 peptide. (*c*) 14-3-3 isoform sequence alignment.

The binding of the SHN3 peptides to 14-3-3 proteins was first characterized using a fluorescence polarization assay exploiting a fluorescein isothiocyanate (FITC) version of the peptides. 14-3-3 proteins, in their full-length versions, were titrated against a fixed concentration of peptides in order to generate polarization attributable to a binding event (Figs. 1 ▸ *a* and 1 ▸ *b*). All of the 14-3-3 isoforms bound to the SHN3pS542 peptide, generating full titration curves, from which it was possible to calculate affinities in the low micromolar range: from 0.5 to 2 µ*M* (Fig. 1 ▸ *a*). 14-3-3σ, however, generated a curve which underwent a considerable left shift towards higher affinities compared with the other isoforms, generating a nanomolar affinity: 26 ± 2 n*M* (Fig. 1 ▸ *a*). In contrast, the SHN3pT869 peptide failed to provide full titration curves as only a modest FP signal was observed at the highest concentrations. It was therefore not possible to calculate *K*_d_ values (Fig. 1 ▸ *b*). The SHN3pT869 peptide can be considered to be a very weak 14-3-3 binder.

Recent work by Gogl *et al.* (2021 ▸) characterized the interaction between 14-3-3 proteins and E6 protein-derived phosphopeptides and reported how different peptides maintain a conserved affinity hierarchy towards 14-3-3 isoforms. 14-3-3 isoforms were ranked into four groups on the basis of the measured affinities (highest to lowest) towards the phosphorylated peptides studied: γ > η > ζ/τ/β > ɛ/σ. Interestingly, the same trend is observed for many other 14-3-3 targets reported in the literature (Stevers *et al.*, 2016 ▸; Soini, Leysen, Davis & Ottmann, 2020 ▸; Soini, Leysen, Davis, Westwood *et al.*, 2020 ▸; Centorrino *et al.*, 2018 ▸). The FP assay conducted on the SHN3pS542 peptide also revealed the same trend, except for 14-3-3η, which showed a higher affinity compared with 14-3-3γ, although the calculated values were remarkably similar: 0.69 ± 0.06 µ*M* for 14-3-3η and 0.78 ± 0.05 µ*M* for 14-3-3γ. However, we unexpectedly discovered an exception to this rule, with 14-3-3σ found to be the highest affinity isoform, binding the SHN3pS542 peptide with low-nanomolar affinity. The binding of SHN3pS542 to 14-3-3 was further characterized by ITC. The peptide was tested against the 14-3-3γ isoform, which was taken as a reference for all of the other isoforms. The estimated *K*_d_ for this assay was 0.69 ± 0.15 µ*M*, which matched that estimated with the FP assay: 0.78 ± 0.05 µ*M* (Figs. 1 ▸ *a* and 2 ▸ *a*).

### 3.3. X-ray structural characterization of the SHN3pS542 and SHN3pT869 peptides in complex with 14-3-3σ   

To structurally elucidate the binding of the SHN3pS542 and the SHN3pT869 peptides, they were crystallized with 14-3-3σ. Despite SHN3pT869 being a very weak binder of 14-3-3 proteins, X-ray crystallographic experiments with this peptide were implemented. The 14-3-3σ isoform was chosen since crystallization conditions are known that readily produce crystals independent of the peptides being used. Also, we hoped that it could provide insight into why the SHN3pS542 peptide bound more strongly to this isoform. High-resolution structures of 14-3-3σ in complex with each peptide were obtained. The data-collection and refinement statistics for the solved structures are reported in Table 2 ▸. For both structures, the asymmetric unit is composed of one 14-3-3σ monomer bound to one copy of the peptide (Figs. 3 ▸ *a* and 3 ▸ *b*). The peptide sequences bound to 14-3-3σ were modelled in the 2*F*_o_ − *F*_c_ map contoured at σ = 1, which allowed nine residues out of 12 to be built for both SHN3pS542 and SHN3pT869 (Figs. 3 ▸ *e* and 3 ▸ *f*). The two peptides bound to the canonical amphipathic 14-3-3 groove employ the phosphorylated Ser542 and Thr869 as the major anchor points. These generate polar contacts with Arg56, Arg129 and Tyr130 from 14-3-3σ (Figs. 3 ▸ *c* and 3 ▸ *d*). Besides the phosphorylated Ser542 and Thr869, the peptides interact with different residues on 14-3-3σ. Polar contacts are established between Glu872 and Glu870 of SHN3pT869 and Asn50, Lys122, Lys49 and Asn175 on the C-terminal side of the peptide and between Asp868 and Pro867 of SHN3pT869 and Asn226 on the N-terminal side of the peptide (Fig. 3 ▸ *d*). In contrast, polar contacts are established between His541 and Ser540 of SHN3pS542 and Asn226, Trp230 and Glu182 on the N-terminal side of the peptide (Fig. 3 ▸ *c*). The C-terminal side sees the SHN3pS542 peptide extending over the entire 14-3-3σ cavity, generating contacts between Ala547, Ala546, Ser545 and Met543 with Asn42, Ser45 and Lys122 on 14-3-3σ.

**Figure 2:**
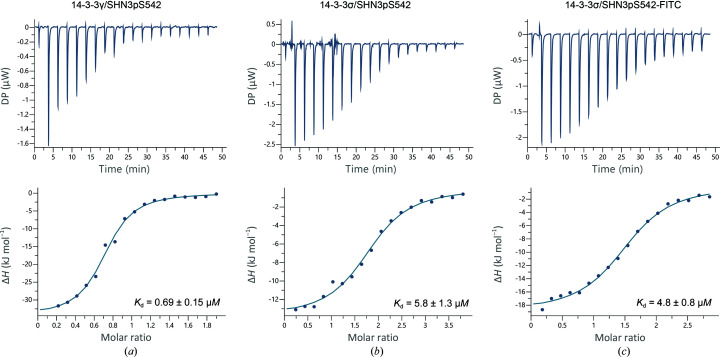
Isothermal titration calorimetry assays of 14-3-3 proteins with SHN3pS542. (*a*) SHN3pS542 peptide titrated against 14-3-3γ. (*b*) SHN3pS542 peptide titrated against 14-3-3σ under reducing conditions. (*c*) SHN3pS542-FITC peptide titrated against 14-3-3σ under reducing conditions.

**Figure 3:**
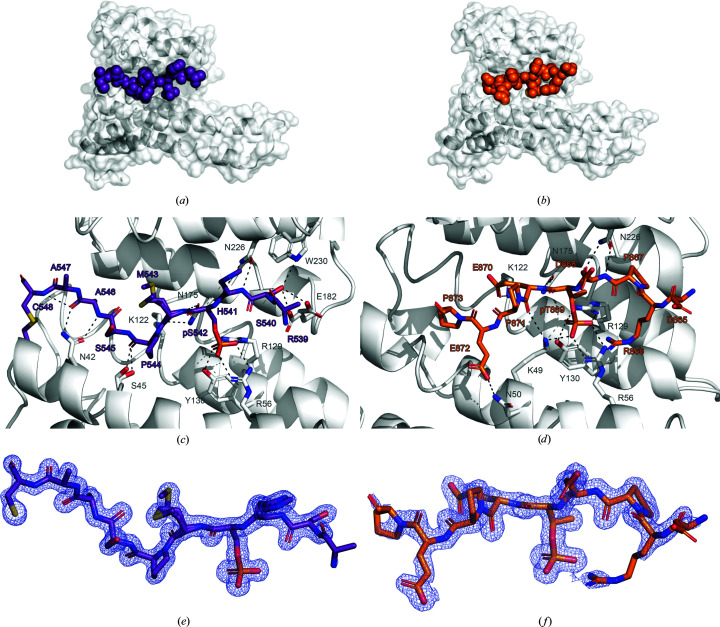
Crystal structures of 14-3-3σ in complex with the phosphorylated peptides SHN3pS542 and SHN3pT869. (*a*, *b*) Surface representations of the asymmetric units of the 14-3-3σ–SHN3pS542 and 14-3-3σ–SHN3pT869 crystal structures. The 14-3-3σ monomer is represented as a white surface with transparency at 60% and as a white cartoon highlighting the secondary structure of 14-3-3 proteins: nine α-helices and loops that connect them. The SHN3pS542 peptide is represented as purple spheres and the SHN3pT869 peptide is represented as orange spheres. (*c*, *d*) Stick representation of the SHN3pS542 peptide (purple) and SHN3pT869 peptide (orange) bound to the 14-3-3σ amphipathic binding groove. Polar bonds are represented as dotted black lines. (*e*, *f*) Stick representation of the SHN3pS542 peptide (purple) and the SHN3pT869 peptide (orange) with their 2*F*_o_ − *F*_c_ maps contoured at σ = 1. The side chain of Arg539 of the SHN3pS542 peptide has not been modelled due to the lack of electron density in this area.

**Figure 4:**
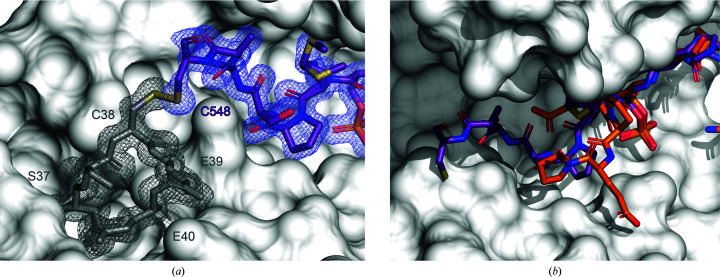
Magnification of the SHN3pS542 and SHN3pT869 peptides bound to 14-3-3σ. (*a*) The SHN3pS542 peptide is represented as purple sticks bound covalently through a disulfide bond to Cys38 of 14-3-3σ. 14-3-3σ is represented as a white surface and its Ser37, Cys38, Glu39 and Glu40 residues as grey sticks. The 2*F*_o_ −* F*_c_ map countered at σ = 1 is coloured blue for SHN3pS542 and grey for 14-3-3σ, suggesting the presence of a disulfide bond between Cys38 and Cys548. (*b*) Superposition of the SHN3pS542 and SHN3pT869 peptides bound to 14-3-3σ. The SHN3pS542 peptide (purple) occupies the entire 14-3-3σ groove, whereas the two consecutive prolines in SHN3pT869 cause the peptide to turn away from the 14-3-3 groove.

14-3-3 proteins often bind to pSer/pThr located in intrinsically disordered regions, inducing a disorder-to-order transition effect on their binding partners (Bustos & Iglesias, 2006 ▸; Sluchanko & Bustos, 2019 ▸). Therefore, in 14-3-3–phosphopeptide crystal structures, usually only three or four amino acids can be modelled on each side of the central pSer/Thr anchoring residue. However, it was possible to model the entire sequence of the SHN3pS542 peptide from pSer542 to Cys548 (Fig. 3 ▸ *c*). The 2*F*_o_ − *F*_c_ map strongly suggests that a disulfide bond is formed between Cys548 on SHN3pS542 and Cys38 on 14-3-3σ. This keeps the peptide rigidly docked close to the 14-3-3 surface, explaining why more of the peptide could be modelled in the electron density. It also explains the observed higher affinity of the SHN3pS542 peptide for the 14-3-3σ isoform, as the other isoforms do not have a cysteine at the corresponding position in their primary sequence (Fig. 1 ▸ *c*). If we compare the two SHN3 peptides bound to 14-3-3σ it is possible to notice how the covalent disulfide bond acts on the orientation that the SHN3pS542 peptide takes within the 14-3-3 binding groove (Fig. 4 ▸ *b*). The covalent bond in fact forces the peptide to occupy the entire length of the cavity. This evidence was found to be in total agreement with what is observed in the FP assay, which showed a 100-fold increase in binding affinity for 14-3-3σ compared with the other 14-3-3 isoforms (Fig. 1 ▸ *a*). 14-3-3 proteins possess a high degree of sequence similarity among the isoforms. 14-3-3σ and 14-3-3γ are 65% identical, but the degree of similarity can be as high as 87% for the 14-3-3η and 14-3-3γ isoforms (UniProt). However, Cys38 is present only in the 14-3-3σ isoform, which explains why the covalent disulfide bond could only form with this specific isoform (Fig. 1 ▸ *c*). As discussed above, the nanomolar affinity of SHN3pS542 for 14-3-3σ was a surprising exception compared with the 14-3-3 hierarchy of binding affinity, where 14-3-3σ is normally the weakest binder to phosphorylated peptides (Gogl *et al.*, 2021 ▸). After the observation that the SHN3pS542 peptide binds covalently to 14-3-3σ in the crystal structure, we tested it in an ITC assay under reducing conditions (Fig. 2 ▸). The peptide was tested against the 14-3-3σ isoform in its acetylated version, as used in crystallography, and in its FITC-labelled form, as used in the FP assay. As expected, the *K*_d_ values determined for the σ isoform were significantly weaker under reducing conditions: 5.8 ± 1.3 µ*M* for the acetylated peptide (Fig. 2 ▸ *b*) and 4.8 ± 0.8 µ*M* for the FITC-labelled form (Fig. 2 ▸ *c*). This shows that in the absence of the disulfide bond both peptides had *K*_d_ values in the same affinity range as observed by FP for the other 14-3-3 isoforms (Fig. 1 ▸ *a*). Moreover, the *K*_d_ determination of the SHN3pS542 peptide under reducing conditions restored the affinity hierarchy, with 14-3-3σ as the weakest isoform binder (Gogl *et al.*, 2021 ▸).

In contrast, the SHN3pT869 peptide adopts the typical conformation, with the proline in the +2 position from pThr869 inducing a turn away from the 14-3-3 binding groove (Yaffe *et al.*, 1997 ▸; Rittinger *et al.*, 1999 ▸).

A closer look at the peptide sequences highlights the conclusion that the SHN3pS542 peptide is a pure mode I binder, while the proline at position −2 from the pThr869 site replaces the ideal hydroxylic/aromatic residue. Despite the discovery of exceptions to the canonical binding motifs (Johnson *et al.*, 2010 ▸), the presence of an aliphatic residue may be the cause of the weak affinity of the SHN3pT869 peptide for 14-3-3 proteins. Moreover, Gogl and coworkers described how the binding of phosphopeptides to 14-3-3 proteins is governed by subtle sequence variations that modulate the intermolecular and intramolecular contacts of 14-3-3–peptide complexes and intramolecular contacts of the peptide in its unbound state (Gogl *et al.*, 2021 ▸). Charge distribution caused by specific residues on the unbound form of the peptides might be responsible for the formation of charge clamps which can hamper the binding to 14-3-3. Despite the highly conserved nature of 14-3-3 recognition motifs, subtle changes in these sequences have been proved to be sufficient to generate a ∼40 000-fold difference in affinity between the weakest and stronger 14-3-3 binders that are reported in the literature (Gogl *et al.*, 2021 ▸).

One of the proposed mechanisms that 14-3-3 proteins utilize to dock onto binding partners is the so-called gatekeeper model. In this model, it is proposed that at first a stronger site interacts with one binding groove on 14-3-3. This would then cause the second weaker site to be in close proximity to bind to the second 14-3-3 binding groove, taking advantage of the avidity effect caused by the first binding event (Molzan & Ottmann, 2012 ▸; Obsil *et al.*, 2003 ▸; Kostelecky *et al.*, 2009 ▸; Yaffe, 2002 ▸). Having two sites bound to 14-3-3 simultaneously improves the affinity of the whole system. This mechanism of action highlights the fact that there could be many 14-3-3 sites situated on binding partners that would not necessarily be revealed by biophysical techniques such as FP and ITC. They would in fact need to be linked to a first stronger site. The fact that the SHN3pT869 peptide did not show a comparable affinity for 14-3-3 proteins to SHN3pS452 could theoretically be explained by the fact that it is part of a more complicated mode of binding such as the gatekeeper model. Considering this, it is important to be aware that more 14-3-3 binding sites beyond those studied in this paper could be present on SHN3 and be fundamental for the formation of such complexes *in vivo.*

## 4. Conclusions   

In this work, we show that in the form of 12-mer peptides, the SHN3pS542 and SHN3pT869 sites interact with 14-3-3 proteins *in vitro* and utilize the canonical 14-3-3 binding groove. FP and ITC assays supported the binding of SHN3pS542 to all 14-3-3 proteins, whereas the SHN3pT869 interaction was only confirmed under the artificially high protein concentrations required to obtain the crystal structure. Significant differences in binding affinity were observed and the SHN3pS542 peptide established a disulfide bond with Cys38, which is present only in the 14-3-3σ isoform. This is the first observation of this phenomenon, and it will be interesting to see whether this also occurs within the cellular environment and with full-length SHN3. A covalent interaction may define a new biological function for 14-3-3σ, for example a more permanent 3D structural determinant for disordered proteins such as SHN3.

Future studies to explore this hypothesis will be expanded to include known, physiologically relevant 14-3-3 interactions with binding sites that have a similarly placed cysteine. On examination of the current ‘gold standard’ list of 14-3-3 binding sites (Madeira *et al.*, 2015 ▸), we note this includes one centred around Ser346 of PKCɛ, which controls kinase activation (Kostelecky *et al.*, 2009 ▸). PKCɛ ablation in rats and mice protects against diet-induced glucose intolerance or liver insulin resistance (Schmitz-Peiffer, 2020 ▸), so it would be interesting to investigate whether 14-3-3σ has a specialized role in metabolic control by virtue of its Cys38 residue. The presence of a proximal reactive cysteine residue may also allow the use of covalent chemical probes to modulate any observed biological functionality (Sijbesma *et al.*, 2019 ▸, 2020 ▸).

In conclusion, the findings presented in this paper represent a starting point for investigating both SHN3–14-3-3 inter­actions and the possible role of interprotein disulfide bonding in 14-3-3σ interactions.

## Supplementary Material

PDB reference: 14-3-3σ–SHN3pS542, 7b13

PDB reference: 14-3-3σ–SHN3pT869, 7b15

Supplementary Figures. DOI: 10.1107/S2053230X21006658/ag5041sup1.pdf

## Figures and Tables

**Table 1:** Putative 14-3-3 binding sites identified on SHN3 by the *14-3-3-Pred* web server

pSer/pThr site	Sequence	Conservation†	Proline +2	Mode I/II‡
399	YFSRSE(pSer)AEQQVS	—	—	Hybrid
490	VKPRRS(pSer)LSRRSM	—	—	Hybrid
542	PLLRSH(pSer)MPSAAC	Y	Y	I (Arg −3; Pro +2)
793	GKERRT(pThr)SKEISV	—	—	Hybrid
794	KERRTT(pSer)KEISVI	—	—	Hybrid
869	EPDRPD(pThr)EPEPPP	Y	Y	I (Arg −3; Pro +2)
892	WPQRSQ(pThr)LAQLPA	—	—	Hybrid
933	PLSRSP(pSer)QESNVS	—	—	Hybrid
948	GSSRSA(pSer)FERDDH	—	—	Hybrid
993	EMRRSA(pSer)EQSPNV	—	—	Hybrid
1012	TETRSK(pSer)FDYGSL	—	—	Hybrid
1050	FLVRQA(pSer)LSRPPE	—	—	Hybrid
1401	YLRVPV(pThr)LPERKG	—	Y	II (Arg −4; Pro +2)
1625	HADRRS(pSer)VYAGWC	—	—	Hybrid
1894	HALRAD(pSer)SPILGP	—	Y	I (Arg −3; Pro +2)
1986	PLARKH(pSer)LTKNDS	—	—	Hybrid
2339	ESPRAP(pThr)NPEPSA	—	Y	I (Arg −3; Pro +2)
2354	PLDRSS(pSer)VGCLAE	—	—	Hybrid

† Conservation was tested against mouse SHN3.

‡ Mode I, RS*X*pS/pT*X*P; mode II, R*X*Y/F*X*pS/pT*X*P.

**Table 2:** Data-collection and refinement statistics for the 14-3-3σ–SHN3pS542 and 14-3-3σ–SHN3pT869 crystal structures Values in parentheses are for the highest resolution shell.

	14-3-3σ–SHN3pS542	14-3-3σ–SHN3pT869
Data collection		
Space group	*C*222_1_	*C*222_1_
*a*, *b*, *c* (Å)	82.76, 112.83, 62.93	83.29, 113.63, 63.20
Resolution (Å)	28.46–1.37 (7.52–1.37)	56.72–1.59 (8.68–1.59)
*R*_merge_ (%)	0.050 (0.32)	0.042 (0.15)
〈*I*/σ(*I*)〉	14.1 (3.2)	19.3 (5.6)
Completeness (%)	99.9 (99.9)	99.8 (99.7)
Multiplicity	5.2 (3.4)	5.2 (3.5)
CC_1/2_	0.99 (0.84)	0.99 (0.96)
Refinement		
Resolution (Å)	28.46–1.37	56.72–1.59
No. of reflections	61661	40935
*R*_work_/*R*_free_	0.14/0.16	0.13/0.15
No. of atoms		
Protein	2124	2121
Water	412	394
*B* factors (Å^2^)		
Protein	17.89	19.66
Water	34.35	35.67
R.m.s.d.		
Bond lengths (Å)	0.018	0.011
Bond angles (°)	1.56	1.02
Ramachandran statistics (%)		
Favoured	97.91	98.32
Allowed	2.09	1.68
Outliers	0.00	0.00

## References

[bb2] Aitken, A. (2006). *Semin. Cancer Biol.* **16**, 162–172. 10.1016/j.semcancer.2006.03.005 16678438

[bb3] Ballone, A., Centorrino, F., Wolter, M. & Ottmann, C. (2018). *J. Struct. Biol.* **202**, 210–215. 10.1016/j.jsb.2018.01.011 29408703

[bb4] Bustos, D. M. & Iglesias, A. A. (2006). *Proteins*, **63**, 35–42. 10.1002/prot.20888 16444738

[bb5] Centorrino, F., Ballone, A., Wolter, M. & Ottmann, C. (2018). *FEBS Lett.* **592**, 1211–1220. 10.1002/1873-3468.13017 29473952

[bb6] Cornell, B. & Toyo-oka, K. (2017). *Front. Mol. Neurosci.* **10**, 318. 10.3389/fnmol.2017.00318 PMC5643407 29075177

[bb7] Emsley, P., Lohkamp, B., Scott, W. G. & Cowtan, K. (2010). *Acta Cryst.* D**66**, 486–501. 10.1107/S0907444910007493 PMC2852313 20383002

[bb8] Evans, P. R. (2011). *Acta Cryst.* D**67**, 282–292. 10.1107/S090744491003982X PMC3069743 21460446

[bb9] Gogl, G., Tugaeva, K. V., Eberling, P., Kostmann, C., Trave, G. & Sluchanko, N. N. (2021). *Nat. Commun.* **12**, 1677. 10.1038/s41467-021-21908-8 PMC7961048 33723253

[bb10] Harada, S. & Rodan, G. A. (2003). *Nature*, **423**, 349–355. 10.1038/nature01660 12748654

[bb11] Hermeking, H. & Benzinger, A. (2006). *Semin. Cancer Biol.* **16**, 183–192. 10.1016/j.semcancer.2006.03.002 16697662

[bb12] Johnson, C., Crowther, S., Stafford, M. J., Campbell, D. G., Toth, R. & MacKintosh, C. (2010). *Biochem. J.* **427**, 69–78. 10.1042/BJ20091834 PMC2860806 20141511

[bb13] Johnson, C., Tinti, M., Wood, N. T., Campbell, D. G., Toth, R., Dubois, F., Geraghty, K. M., Wong, B. H., Brown, L. J., Tyler, J., Gernez, A., Chen, S., Synowsky, S. & MacKintosh, C. (2011). *Mol. Cell. Proteomics*, **10**, M110.005751. 10.1074/mcp.M110.005751 PMC3205853 21725060

[bb14] Jones, D. C., Wein, M. N. & Glimcher, L. H. (2007). *Adv. Exp. Med. Biol.* **602**, 1–13. 10.1007/978-0-387-72009-8_1 17966382

[bb15] Kabsch, W. (2010). *Acta Cryst.* D**66**, 133–144. 10.1107/S0907444909047374 PMC2815666 20124693

[bb16] Kim, J.-M., Yang, Y.-S., Park, K. H., Oh, H., Greenblatt, M. B. & Shim, J.-H. (2019). *Int. J. Mol. Sci.* **20**, 1803. 10.3390/ijms20081803 PMC6514701 31013682

[bb17] Kostelecky, B., Saurin, A. T., Purkiss, A., Parker, P. J. & McDonald, N. Q. (2009). *EMBO Rep.* **10**, 983–989. 10.1038/embor.2009.150 PMC2750047 19662078

[bb1] Liebschner, D., Afonine, P. V., Baker, M. L., Bunkóczi, G., Chen, V. B., Croll, T. I., Hintze, B., Hung, L.-W., Jain, S., McCoy, A. J., Moriarty, N. W., Oeffner, R. D., Poon, B. K., Prisant, M. G., Read, R. J., Richardson, J. S., Richardson, D. C., Sammito, M. D., Sobolev, O. V., Stockwell, D. H., Terwilliger, T. C., Urzhumtsev, A. G., Videau, L. L., Williams, C. J. & Adams, P. D. (2019). *Acta Cryst.* D**75**, 861–877. 10.1107/S2059798319011471 PMC6778852 31588918

[bb18] Mackintosh, C. (2004). *Biochem. J.* **381**, 329–342. 10.1042/BJ20031332 PMC1133837 15167810

[bb19] Madeira, F., Tinti, M., Murugesan, G., Berrett, E., Stafford, M., Toth, R., Cole, C., MacKintosh, C. & Barton, G. J. (2015). *Bioinformatics*, **31**, 2276–2283. 10.1093/bioinformatics/btv133 PMC4495292 25735772

[bb20] McCoy, A. J., Grosse-Kunstleve, R. W., Adams, P. D., Winn, M. D., Storoni, L. C. & Read, R. J. (2007). *J. Appl. Cryst.* **40**, 658–674. 10.1107/S0021889807021206 PMC2483472 19461840

[bb21] Molzan, M. & Ottmann, C. (2012). *J. Mol. Biol.* **423**, 486–495. 10.1016/j.jmb.2012.08.009 22922483

[bb22] Obsil, T., Ghirlando, R., Anderson, D. E., Hickman, A. B. & Dyda, F. (2003). *Biochemistry*, **42**, 15264–15272. 10.1021/bi0352724 14690436

[bb23] Obsil, T. & Obsilova, V. (2011). *Semin. Cell Dev. Biol.* **22**, 663–672. 10.1016/j.semcdb.2011.09.001 21920446

[bb24] Rittinger, K., Budman, J., Xu, J., Volinia, S., Cantley, L. C., Smerdon, S. J., Gamblin, S. J. & Yaffe, M. B. (1999). *Mol. Cell*, **4**, 153–166. 10.1016/s1097-2765(00)80363-9 10488331

[bb25] Rose, R., Rose, M. & Ottmann, C. (2012). *J. Struct. Biol.* **180**, 65–72. 10.1016/j.jsb.2012.05.010 22634725

[bb26] Schmitz-Peiffer, C. (2020). *Trends Endocrinol. Metab.* **31**, 344–356. 10.1016/j.tem.2020.01.016 32305097

[bb27] Schumacher, B., Skwarczynska, M., Rose, R. & Ottmann, C. (2010). *Acta Cryst.* F**66**, 978–984. 10.1107/S1744309110025479 PMC2935210 20823509

[bb28] Shim, J. H., Greenblatt, M. B., Zou, W., Huang, Z., Wein, M. N., Brady, N., Hu, D., Charron, J., Brodkin, H. R., Petsko, G. A., Zaller, D., Zhai, B., Gygi, S., Glimcher, L. H. & Jones, D. C. (2013). *J. Clin. Invest.* **123**, 4010–4022. 10.1172/JCI69443 PMC3754267 23945236

[bb29] Sijbesma, E., Hallenbeck, K. K., Leysen, S., de Vink, P. J., Skóra, L., Jahnke, W., Brunsveld, L., Arkin, M. R. & Ottmann, C. (2019). *J. Am. Chem. Soc.* **141**, 3524–3531. 10.1021/jacs.8b11658 30707565

[bb30] Sijbesma, E., Somsen, B. A., Miley, G. P., Leijten-van de Gevel, I. A., Brunsveld, L., Arkin, M. R. & Ottmann, C. (2020). *ACS Chem. Biol.* **15**, 3143–3148. 10.1021/acschembio.0c00646 PMC7754187 33196173

[bb31] Sluchanko, N. N. (2018). *J. Mol. Biol.* **430**, 20–26. 10.1016/j.jmb.2017.11.010 29180038

[bb32] Sluchanko, N. N. & Bustos, D. M. (2019). *Prog. Mol. Biol. Transl. Sci.* **166**, 19–61. 10.1016/bs.pmbts.2019.03.007 31521232

[bb33] Sluchanko, N. N. & Gusev, N. B. (2010). *Biochemistry (Mosc.)*, **75**, 1528–1546. 10.1134/s0006297910130031 21417993

[bb34] Soini, L., Leysen, S., Davis, J. & Ottmann, C. (2020). *J. Struct. Biol.* **212**, 107662. 10.1016/j.jsb.2020.107662 33176192

[bb35] Soini, L., Leysen, S., Davis, J., Westwood, M. & Ottmann, C. (2021). *FEBS Lett.* **595**, 404–414. 10.1002/1873-3468.13993 33159816

[bb36] Stevers, L. M., Lam, C. V., Leysen, S. F., Meijer, F. A., van Scheppingen, D. S., de Vries, R. M., Carlile, G. W., Milroy, L. G., Thomas, D. Y., Brunsveld, L. & Ottmann, C. (2016). *Proc. Natl Acad. Sci. USA*, **113**, E1152–E1161. 10.1073/pnas.1516631113 PMC4780605 26888287

[bb37] Winn, M. D., Ballard, C. C., Cowtan, K. D., Dodson, E. J., Emsley, P., Evans, P. R., Keegan, R. M., Krissinel, E. B., Leslie, A. G. W., McCoy, A., McNicholas, S. J., Murshudov, G. N., Pannu, N. S., Potterton, E. A., Powell, H. R., Read, R. J., Vagin, A. & Wilson, K. S. (2011). *Acta Cryst.* D**67**, 235–242. 10.1107/S0907444910045749 PMC3069738 21460441

[bb38] Yaffe, M. B. (2002). *FEBS Lett.* **513**, 53–57. 10.1016/s0014-5793(01)03288-4 11911880

[bb39] Yaffe, M. B., Rittinger, K., Volinia, S., Caron, P. R., Aitken, A., Leffers, H., Gamblin, S. J., Smerdon, S. J. & Cantley, L. C. (1997). *Cell*, **91**, 961–971. 10.1016/s0092-8674(00)80487-0 9428519

[bb40] Yang, Y.-S., Xie, J., Wang, D., Kim, J.-M., Tai, P. W. L., Gravallese, E., Gao, G. & Shim, J.-H. (2019). *Nat. Commun.* **10**, 2958. 10.1038/s41467-019-10809-6 PMC6609711 31273195

