# Supplementary material for: The identification and structural analysis of potential 14-3-3 interaction sites on the bone regulator protein Schnurri-3
Source: Acta Crystallogr F Struct Biol Commun. 2021 Jul 28;77(Pt 8):254–61. doi: 10.1107/S2053230X21006658 (PMC8329713; doi:10.1107/S2053230X21006658)
Supplement: Supplementary file 1 [file f-77-00254-sup1.pdf]

STRUCTURAL BIOLOGY
COMMUNICATIONS

Volume 77 (2021)

Supporting information for article:

The identification and structural analysis of potential 14-3-3 interaction sites on the bone regulator protein Schnurri-3

Lorenzo Soini, Seppe Leysen, Tom Crabbe, Jeremy Davis and Christian Ottmann

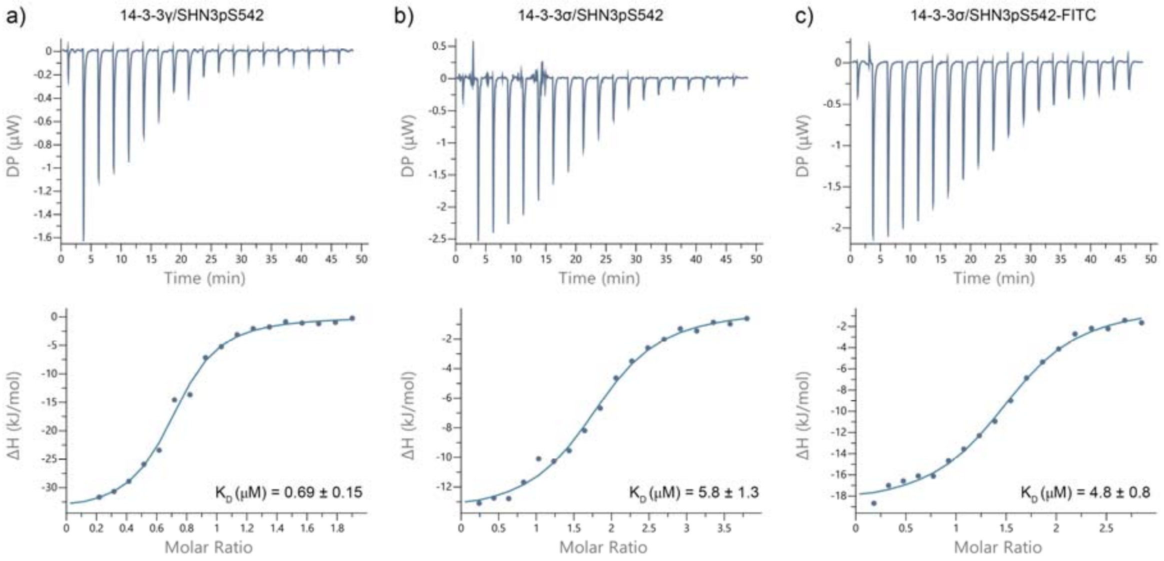

Figure S1 Isothermal Titration Calorimetry assays of 14-3-3 proteins with SHN3pS542. a) SHN3pS542 peptide titrated over 14-3-3γ. b) SHN3pS542 peptide titrated over 14-3-3σ under reducing conditions. c) SHN3pS542-FITC peptide titrated over 14-3-3σ under reducing conditions.

```
                                              ... -38- ...
P31947|σ --MERASLIQKAKLAEQAERYEDMAAFMKGAVEKGEELSCEERNLLSVAYKNVVGGQRAA ... 58
P61981|γ -MVDREQLVQKARLAEQAERYDDMAAAMKNVTELNEPLSNEERNLLSVAYKNVVGARRSS ... 59
Q04917|η -MGDREQLLQRARLAEQAERYDDMASAMKAVTELNEPLSNEDRNLLSVAYKNVVGARRSS ... 59
P31946|β MTMDKSELVQKAKLAEQAERYDDMAAAMKAVTEQGHELSNEERNLLSVAYKNVVGARRSS ... 60
P27348|τ --MEKTELIQKAKLAEQAERYDDMATCMKAVTEQGAELSNEERNLLSVAYKNVVGGRRSA ... 58
P62258|ϵ -MDDREDLVYQAKLAEQAERYDEMVESMKKVAGMDVELTVEERNLLSVAYKNVIGARRAS ... 59
P63104|ζ --MDKNELVQKAKLAEQAERYDDMAACMKSVTEQGAELSNEERNLLSVAYKNVVGARRSS ... 58
```

Figure S2 Alignment of the initial portion of the sequence of all 14-3-3 isoforms up to the first 58/59/60 residues. Homologues residues are highlighted in green and the Cys38 present only in the 14-3-3σ isoform is highlighted in yellow.
